# The critical role of m^6^A methylation in the pathogenesis of Graves' ophthalmopathy

**DOI:** 10.1186/s40662-020-00221-3

**Published:** 2020-12-01

**Authors:** Li Zhu, Siyan Li, Shikun He, Qizhe Tong, Lejin Wang, Xiaohua Li, Xi Wu, Qingyu Meng, Enzhong Jin, Chuan Zhang, Tianyuan Li, Ningda Xu, Lvzhen Huang, Yi Wang, Mingwei Zhao

**Affiliations:** 1 Department of Ophthalmology, Peking University People’s Hospital, Eye Diseases and Optometry Institute, Beijing Key Laboratory of Diagnosis and Therapy of Retinal and Choroid Diseases, College of Optometry, Peking University Health Science Center, Xizhimen South Street 11, Xi Cheng District, Beijing, 100044 China; 2 Henan Provincial People’s Hospital and Henan Eye Hospital, Zhengzhou, China

**Keywords:** m^6^A methylation, Graves' ophthalmopathy, Pathogenesis, RNA-seq

## Abstract

**Purpose:**

To investigate the role of N6-methyladenosine (m^6^A) RNA modification in the pathogenesis of Graves' ophthalmopathy (GO).

**Methods:**

Surgically excised extraocular muscles from 7 patients with GO and 5 subjects without GO were used. The global m^6^A levels in the specimens were determined using an m^6^A RNA methylation quantification kit. RNA sequencing (RNA-seq) was used to analyze the molecules involved in the regulation of m^6^A RNA methylation and the differential expression of mRNAs between the two groups (4 eyes, respectively). The expression of m^6^A RNA modification genes was evaluated by real-time PCR. The functional implications of the gene alterations between the GO and control specimens were determined by Gene Ontology analysis.

**Results:**

The m^6^A level was significantly increased in the specimens of GO patients compared to the control specimens (*P* < 0.05). The expression of m^6^A methylation regulators, such as WT1 associated protein (WTAP), alkylation repair homolog protein 5 (ALKBH5), E74 like ETS transcription factor 3 (ELF3), YTH N6-methyladenosine RNA binding protein 2 (YTHDF2), YTHDF3 and YTH domain containing 2 (YTHDC2), was significantly upregulated (*P* < 0.05). Gene Ontology enrichment analysis showed that the most highly upregulated genes and biological pathways were related to the immune response and inflammatory processes such as lymphocyte activation, leukocyte differentiation, cytokine production and cytokine-mediated signaling pathways.

**Conclusions:**

Our results suggest that m^6^A methylation may play a critical role in the pathogenesis of GO and that targeting genes that regulate m^6^A methylation may provide a new therapeutic approach for GO.

## Background

Graves' ophthalmopathy (GO), also called thyroid eye disease (TED), is the most significant extrathyroidal manifestation of Graves' disease patients. The clinical manifestations of GO result largely from immune and inflammatory responses within the orbit. The pathological process is recognized to be driven by cellular and humoral immunity and inflammation, which stimulate retroocular fibroblast proliferation, local adipogenesis and the inflammatory response of extraocular muscles (EOMs) and interstitial tissues. GO may lead to impairment of ocular motility and lid retraction, exposure keratopathy, optic nerve compression, or even vision loss [1]. Although GO is widely accepted to be an autoimmune process, the exact mechanism underlying its pathogenesis is still under investigation.

Accumulating evidence suggests that epigenetic factors may be involved in the pathogenesis of Graves' disease (GD). Previous studies showed aberrant DNA methylation in GD patients, and genome-wide DNA methylation analysis identified 132 hyper-methylated and 133 hypo-methylated regions in patients with GD [2]. In addition to aberrant of DNA methylation, alterations in histone modification are also found in peripheral blood mononuclear cells from GD patients. The idea that epigenetic factors contribute to the pathogenesis of GD is further supported by a report showing global abnormalities in histone methylation and histone deacetylation in patients with GD [2, 3]. These results suggest that epigenetic modifications contribute to the pathogenesis of both GD and GO; however, the involvement of RNA methylation in the pathogenesis of GD components, particularly GO, has not been reported.

RNA methylation is an essential post-transcriptional mRNA modification event, and N6-methyladenosine (m^6^A) is the most prevalent and abundant eukaryotic mRNA modification [4]. m^6^A is generated post-transcriptionally by a m^6^A methyltransferase complex (m^6^A “writer”). This writer complex comprises methyltransferase-like 3 (METTL3), methyltransferase-like 14 (METTL14), WT1 associated protein (WTAP), vir like m6A methyltransferase associated (VIRMA) and RNA binding motif protein 15 (RBM15). Removal of methyl groups from the adenosine base at the nitrogen-6 position is carried out by enzymes called m^6^A “erasers”. FTO alpha-ketoglutarate dependent dioxygenase (FTO) and alkylation repair homolog protein 5 (ALKBH5) are two identified m^6^A erasers. Proteins that recognize and bind to m^6^A are called “readers”. Members of the YTH domain protein family act as readers, binding to YT521-B homology domains (e.g., YTHDF1–3 and YTHDC1–2) [5].

Recently, emerging evidence has indicated that m^6^A plays crucial roles in many pathological processes, including tumorigenesis, angiogenesis, tissue degeneration and, importantly, autoimmune and inflammatory responses [6, 7]. m^6^A methylation has been reported to be related to NF-κB and MAPK activation and to the inflammatory response [8, 9]. Furthermore, without m^6^A mediated by METTL3, the balance between CD4+ and regulatory T cells is lost [7, 10]. Silencing METTL3 inhibits inflammatory cytokine (i.e., IL-6) production and the major inflammatory signaling pathways [11]. Collectively, the published data suggest that immune and inflammatory responses are tightly regulated by m^6^A methylation. However, the m^6^A methylation status and its underlying mechanism in the pathogenesis of GO remain unclear. Here, we analyzed the levels of m^6^A and its associated molecules from surgically excised EOMs of GO patients and exotropia patients (control subjects) and used Gene Ontology analysis to assess the relevance of these levels to functional enrichment. To our knowledge, this report is the first to investigate whether m^6^A RNA methylation is altered in specimens from GO patients.

## Materials and methods

### Collection of EOMs

Patients and control subjects were recruited from the Department of Ophthalmology of Peking University People’s Hospital (Beijing, China). The study was approved by the Clinic Institutional Review Board of Peking University People’s Hospital (2019PHB312–01) and complied with the Declaration of Helsinki. Written informed consent was obtained from all patients prior to enrolment in the study. GO was diagnosed based on a history of GD (by orbital specialists at Peking University People’s Hospital). Patients enrolled in the study had moderate to severe proptosis as measured by a Hertel exophthalmometer and confirmed by computed tomography (CT) scan of the orbit. All patients had been in euthyroid status for at least 3 months and had not been treated with orbital radiotherapy. The clinical characteristics of the patients and their medical treatment are shown in Table 1. All surgeries were performed by ophthalmic specialists in the department. EOM specimens, which was the thickest area of the EOM and associated with compression to optic nerve, were collected during orbital decompression surgery; control specimens were obtained from patients undergoing surgery to correct concomitant exotropia. Most specimens were used for clinical histologic analysis, and some were immersed in RNAlater Stabilization Solution and rapidly frozen at − 80 °C for research purposes.

**Table 1:** Clinical characteristics of GO patients

Patients ID	Gender/age	Months with GO	Months with GD	CAS	Clinical subtype	Smoking status	TRAb (UI/l)	TSH (μUI/ml)	FT4 (ng/dl)	Treatment for GD	Treatment for GO		
										Use of ATD (actual)	Cortico-steroids	Cyclos-porine	Surgical
A	F/69	12	26	5	II	–	3.70	4.358	13.1	+	+	–	+
B	F/63	20	43	4	II	–	2.73	3.137	17.93	–	+	–	+
C	F/40	6	10	5	II	+	1.38	1.689	16.36	+	+	–	+
D	F/52	9	9	6	II	–	7.34	0.079	17.33	+	+	+	+
E	M/57	6	20	3	II	+	8.58	2.258	18.03	+	+	–	+
F	M/35	7	30	3	II	–	5.33	2.512	14.23	+	+	–	+
G	F/36	11	13	2	II	–	2.46	0.055	13.38	+	+	–	+

*GO* = Graves' ophthalmopathy; *GD* = Graves' disease; *CAS* = Clinical Activity Score; *TRAb* = antibodies against TSH receptor; *TSH* = thyroid-stimulating hormone; *FT4* = free thyroxine; *ATD* = anti-thyroid drug

### m^6^A RNA quantification

Total RNA was isolated from EOMs using TRIzol reagent (Life Technologies Invitrogen Co., Carlsbad, CA, USA). An EpiQuik™ m^6^A RNA Methylation Quantification Kit (Colorimetric, Epigentek Group, NY, USA) was used to measure the m^6^A content in the total RNA samples according to the manufacturer’s instructions. In brief, the assay wells were coated with 200 ng of RNA. The capture and detection antibodies were then added separately to the assay wells. The m^6^A levels were quantified colorimetrically by reading the absorbance of each well at a wavelength of 450 nm (SpectraMax® 190 Microplate Reader, Molecular Devices, LLC), and the m^6^A contents were then calculated based on the standard curve.

### Library construction for transcriptome sequencing

Sequencing libraries were constructed using an NEBNext® Ultra™ RNA Library Prep Kit from Illumina® (NEB, USA) following the manufacturer’s instructions. Index codes were added to attribute sequences to each sample.

### Mapping of reads to the reference genome

Reference genome and gene model annotation files were downloaded directly from the genome website. The index of the reference genome was built using Hisat2 (v2.0.5) and paired-end clean reads were aligned to the reference genome using Hisat2 as well. We selected Hisat2 as the mapping tool because Hisat2 can generate a database of splice junctions based on the gene model annotation file and can thus provide better mapping than other non-splice mapping tools.

### Quantification of gene expression

Feature Counts v1.5.0-p3 was used to count the number of reads mapped to each gene. For simultaneous consideration of the effects of sequencing depth and gene length on the read count, the Fragments Per Kilobase per Million mapped reads (FPKM) value is currently the most commonly used parameter for estimating gene expression levels. Therefore, the FPKM value of each gene was calculated based on the length of the gene and number of reads mapped to the gene.

### Differential expression analysis

Differential gene expression analysis of the two groups was performed using the DESeq2 R package. DESeq2 provide statistical routines for determining differential expression in digital gene expression data using a model based on the negative binomial distribution [12]. The resulting *P*-values were adjusted using the Benjamini and Hochberg approach for controlling the false discovery rate. Genes with an adjusted P-value of less than 0.05 identified by DESeq2 were considered differentially expressed.

### Clustering and sequencing

Clustering of the index-coded samples was performed in a cBot Cluster Generation System using a TruSeq PE Cluster Kit (v3-cBot-HS, Illumina) according to the manufacturer’s instructions. After cluster generation, the libraries were sequenced on the Illumina NovaSeq platform, and 150-bp paired-end reads were generated.

### Gene ontogeny analysis

Gene ontogeny analysis was applied to explore the possible biological functions of the differentially expressed genes via Metascape. The significant enriched ontology terms and pathways were identified using Fisher’s exact test.

### Validation of differentially expressed mRNAs by RT-qPCR

To verify the RNA-seq analysis results, RT-qPCR was used to validate the expression levels of 12 selected mRNAs in an additional 4 patients. Total RNA was extracted from EOMs as described above and was then reverse transcribed using a ReverTra Ace® qPCR RT Kit (Toyobo, Japan) according to the manufacturer’s instructions. The relative expression levels of the selected mRNAs were determined using SYBR Green® Realtime PCR Master Mix (Toyobo, Japan). Relative gene expression levels were calculated using the 2^-∆∆CT^ method. GAPDH was used as the internal control. All experiments were conducted in triplicates and were repeated thrice. Primer sequences are listed in Table 2.

**Table 2:** Primer sequences for RT-qPCR

Gene	Forward Primer (5′ - 3′)	Reverse Primer (5′ - 3′)
WTAP	TTGTAATGCGACTAGCAACCAA	GCTGGGTCTACCATTGTTGATCT
METTL3	AGATGGGGTAGAAAGCCTCCT	TGGTCAGCATAGGTTACAAGAGT
METTL14	GAACACAGAGCTTAAATCCCCA	TGTCAGCTAAACCTACATCCCTG
FTO	AACACCAGGCTCTTTACGGTC	TGTCCGTTGTAGGATGAACCC
ALKBH5	AGTTCCAGTTCAAGCCTATTCG	TGAGCACAGTCACGCTTCC
HNRNPA2B1	ATTGATGGGAGAGTAGTTGAGCC	AATTCCGCCAACAAACAGCTT
ELF3	GGCCGATGACTTGGTACTGAC	GCTTGCGTCGTACTTGTTCTTC
YTHDF1	ATACCTCACCACCTACGGACA	GTGCTGATAGATGTTGTTCCCC
YTHDF2	GTTGGTAGCGGGTCCATTACT	GGTCTTCAGTTTAGGTTGCTGT
YTHDF3	TCAGAGTAACAGCTATCCACCA	GGTTGTCAGATATGGCATAGGCT
YTHDC1	AACTGGTTTCTAAGCCACTGAGC	GGAGGCACTACTTGATAGACGA
YTHDC2	GGTATCCCCTGCCGTATATTTTG	CTTTCCCGTCTCTCTGCGG
GAPDH	GGAGCGAGATCCCTCCAAAAT	GGCTGTTGTCATACTTCTCATGG

### Statistical analysis

Data are presented as the mean ± standard deviation (SD). Differences between the two groups were analyzed using a two-tailed Student’s t-test. The criterion for statistical significance was *P* < 0.05.

## Results

### Increased level of m^6^A in GO patients

**Fig. 1:**
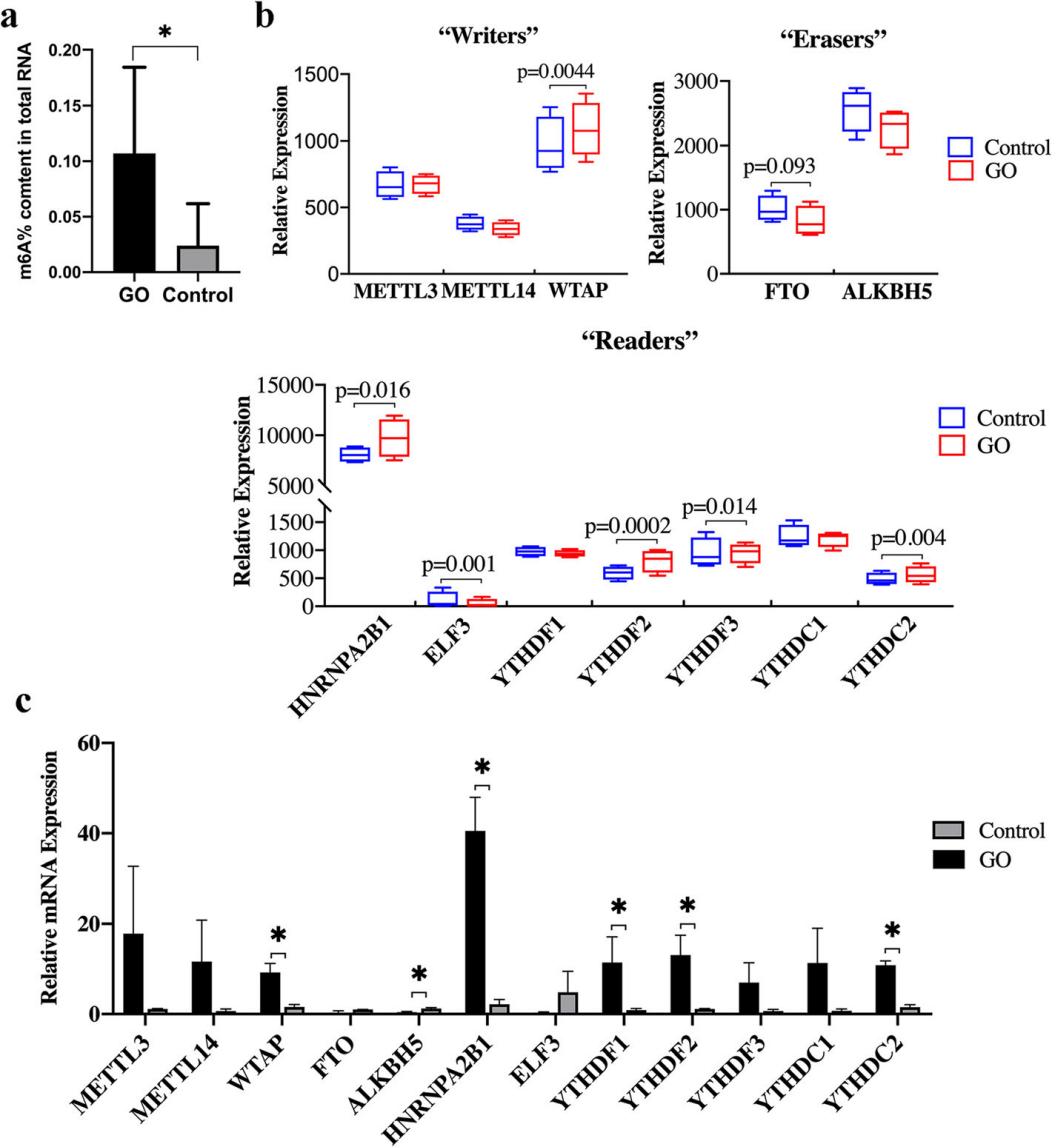
Analysis of m^6^A contents and m6A regulators in GO specimens and controls. **a** The m^6^A contents in total RNA was significantly increased in GO specimens (*n* = 7) compared with controls (*n* = 5) (*p < 0.05). OD reading at 450 nm. **b** m^6^A modification–associated genes were examined by RNA-seq in GO (*n* = 4) extraocular muscles and controls (*n* = 4). The values of the statistics analysis are included in the Figs. **c** Twelve selected m^6^A modification–associated genes were examined in patients with GO (*n* = 7) and controls (*n* = 5) by quantitative PCR and normalized to GAPDH. Student’s t-test was used to assess the differences of each gene between patients in GO and controls. * indicates significant increases of m^6^A mRNA in GO specimens compared with controls (*P* < 0.05)

To explore the potential role of m^6^A modification in the pathogenesis of GO, we extracted total RNA from the EOMs of 7 GO patients and 5 control subjects and then measured the global m^6^A levels in the total RNA samples. Using the colorimetric m^6^A quantification strategy, we found that m^6^A levels were significantly increased in GO tissues compared with control tissues (*P* = 0.03539) (Fig. 1a).

We then hypothesized that the abnormal m^6^A modification level in GO was caused by dysregulation of the key m^6^A methyltransferases and demethylases and their binding proteins (m^6^A methylation readers). To test our hypothesis, we used RNA sequencing (RNA-seq) to measure the expression levels of these genes in 4 pairs of EOMs from patients with GO and control subjects. WTAP was significantly upregulated in GO EOMs compared with control EOMs (*P* < 0.05, Fig. 1b). In contrast, no significant difference in the expression of FTO and ALKBH5 was observed between EOMs from patients with GO and control subjects. Interestingly, three m^6^A readers—ELF3, YTHDF2 and YTHDC2—were significantly increased in GO EOMs compared with control EOMs (*P* < 0.05). To verify the transcriptome sequencing results, we used RT-qPCR to examine the expression of 12 components associated with m^6^A RNA modification (Fig. 1c). The majority of the PCR results were consistent with the RNA-seq results, showing that WTAP, YHDF2 and YTHDC2 but not ALKBH5 and HNRNPA2B1 were significantly upregulated in GO specimens.

### Differentially expressed mRNAs between GO patients and control subjects

**Fig. 2:**
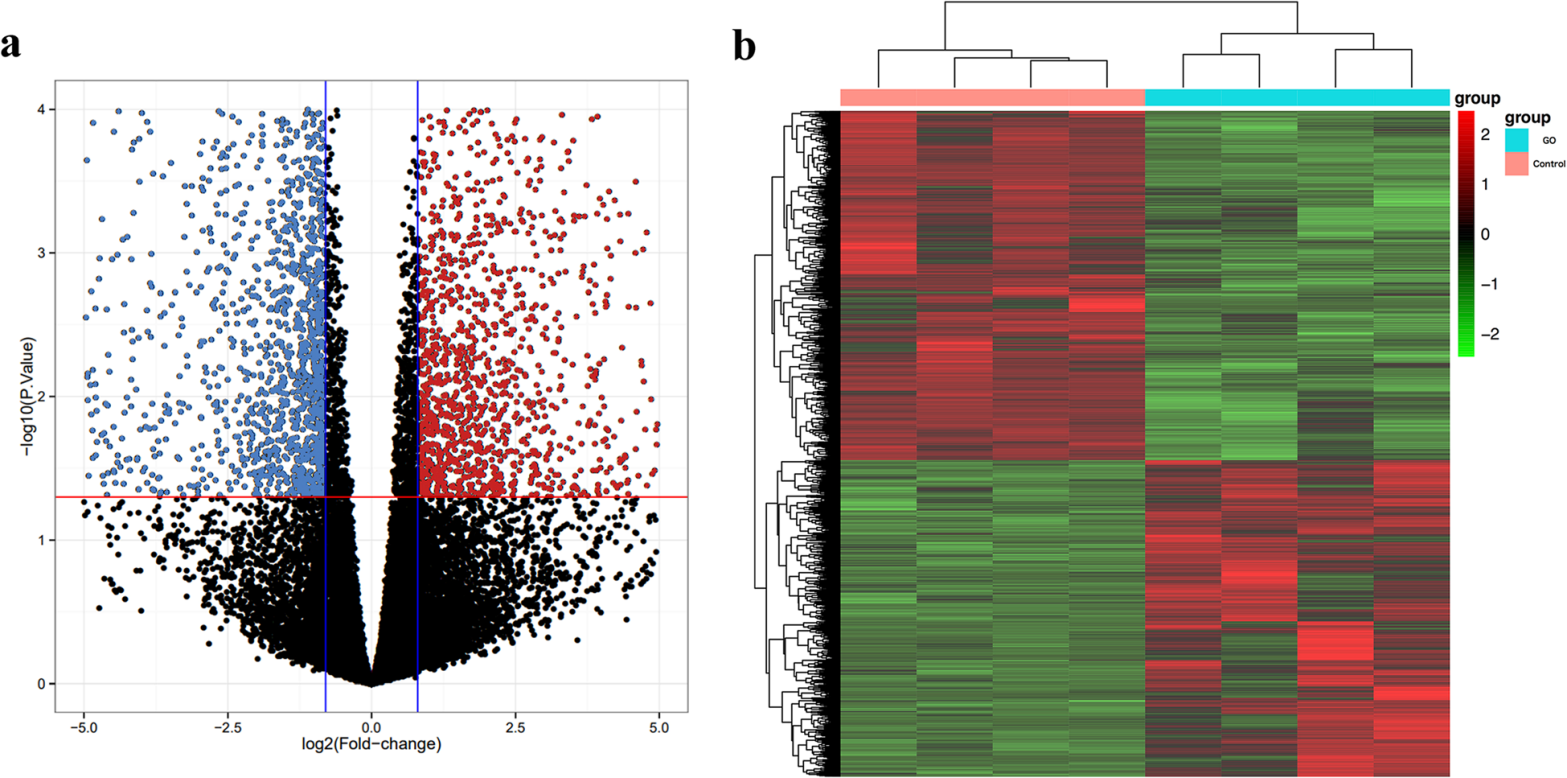
Gene differentiation analysis. **a** Volcano plots of all detected mRNAs in the GO and Control groups. The red dots represent significantly upregulated genes (log2 fold change > 1 and adjusted *P* < 0.05); the blue dots represent significantly downregulated genes (log2 fold change <−1 and adjusted *P* < 0.05); the black dots under the red line indicate that no change of the in the expression levels of those genes were found, and there was no statistical differences of the gene expression levels for those above the red line. **b** Heatmap from the hierarchical clustering analysis showed the differential expressed mRNAs between the two groups. The color scale on the top illustrates the relative expression level of mRNAs across all samples: red denotes mRNA expression greater than the average value, and green denotes mRNA expression lesser than the average value

After measuring the expression of m^6^A modification genes, we analyzed the transcriptome of all genes in a cohort of four specimens from GO patients and four specimens from control subjects. A volcano plot was generated to illustrate the differentially expressed mRNAs in the two groups. The significance threshold was set as a fold change of > 2.0 and adjusted *P* < 0.05. Among the 4393 mRNAs identified, 1374 were upregulated and 1508 were downregulated (GO versus control, Fig. 2a). The heatmap of the hierarchical clustering analysis results shows the expression patterns of distinguishable mRNAs between the two groups (Fig. 2b). These data indicate that the expression of mRNAs significantly changed as a result of GO.

### Gene and pathway enrichment analyses

**Fig. 3:**
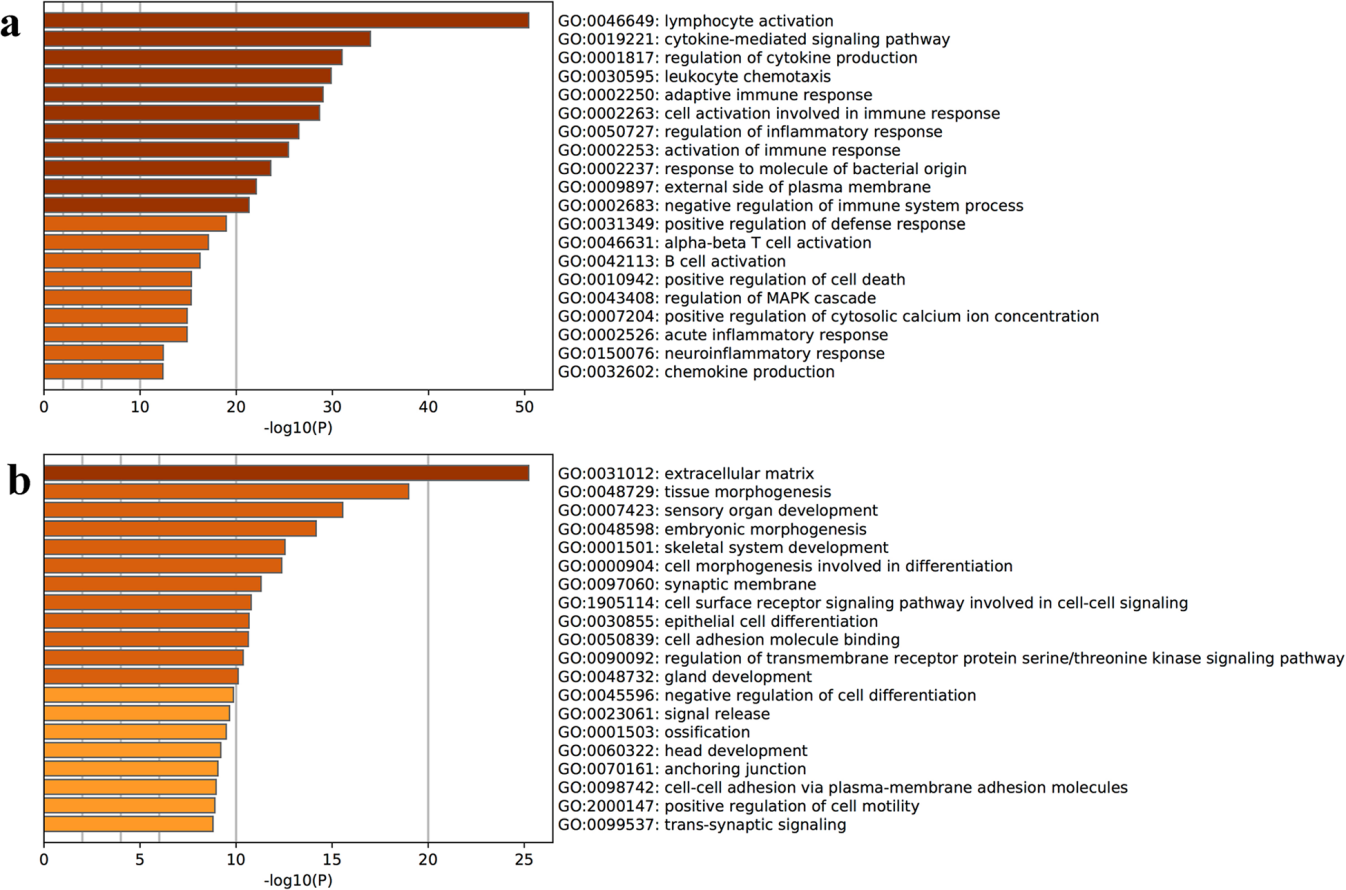
Gene Ontology enrichment analysis. Upregulated (**a**) and downregulated (**b**) genes. The most upregulated genes were related to inflammatory response, while the most downregulated genes were related to tissue development. The darker of the color, the higher expression of the genes

**Fig. 4:**
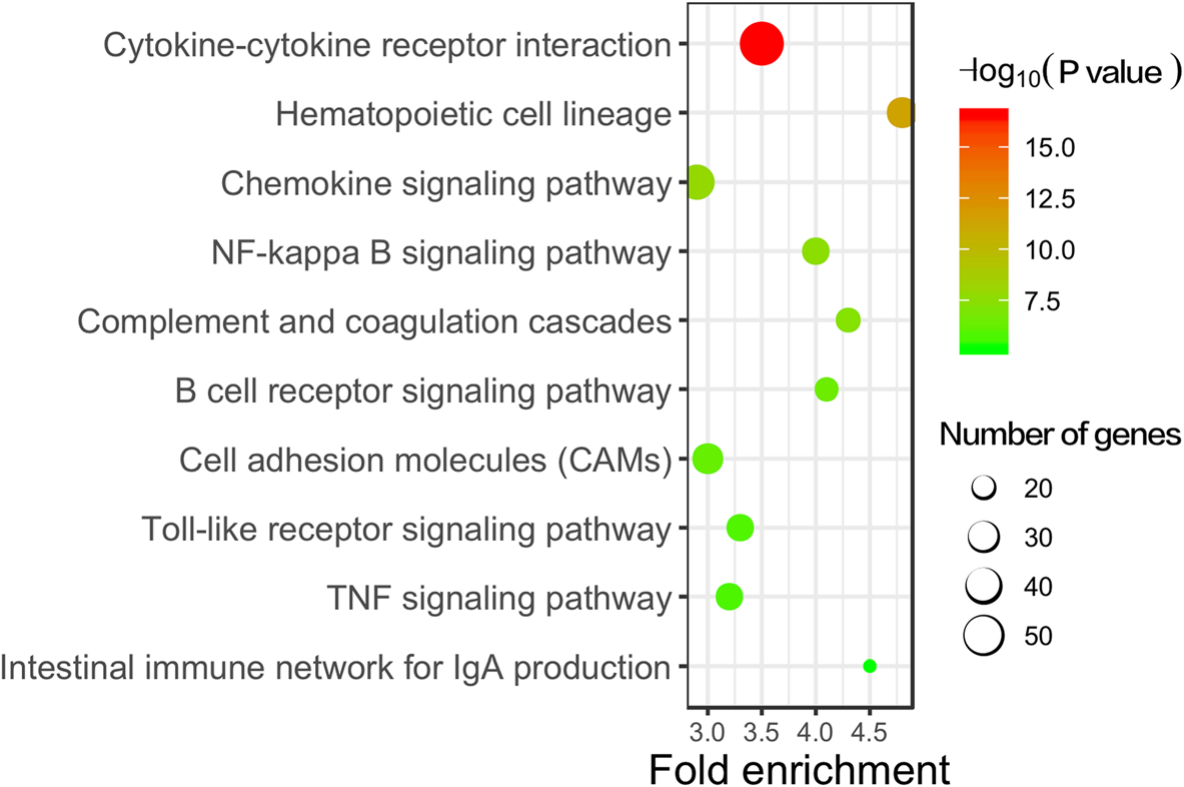
KEGG analysis of the biological pathways. The color scale on the top right shows the relative expression level of the genes and the size of the open circle on the lower right represents the number of genes

Gene enrichment analysis was performed to identify the enrichment of differentially expressed mRNAs and gene products in biological processes, cellular components and molecular functions. Most of the upregulated mRNAs were involved in various immune and inflammatory processes, such as lymphocyte activation, leukocyte differentiation, cytokine production, cytokine-mediated signaling pathways and the adaptive immune response. Conversely, the downregulated differentially expressed genes were associated mainly with tissue morphogenesis, sensory organ development, embryonic morphogenesis and the extracellular matrix (Fig. 3). In addition, pathway analysis was conducted to identify the biological pathways related to the differentially expressed mRNAs. The Kyoto Encyclopedia of Genes and Genomes (KEGG) analysis results indicated that 75 pathways identified in the study significantly differed between the two groups (*P* < 0.01). Consistent with the gene enrichment analysis results, the pathways involved in immune and inflammatory responses, such as cytokine-cytokine interaction, NF-κB signaling, cell receptor signaling, Toll-like receptor signaling and TNF signaling, were the most highly enriched in upregulated genes (Fig. 4).

### Expression of inflammation and immune response-related cytokines

Most of the upregulated genes and signaling pathways were related to inflammation and the immune response. Therefore, the gene expression levels of specific cytokines were measured. IL-6 exhibited the greatest increase in expression; it was upregulated more than 100-fold in GO patients compared with control subjects. In addition, the expression levels of other inflammatory cytokines, such as IL-18, IL-1B, IL-10, TNF, interferon-γ (IFN-γ) and IL-17B, increased more than 2 times relative to those in control subjects, with *P*-values of less than 0.001 (Table 3).

**Table 3:** Expression of cytokines in GO (*n* = 4) extraocular muscles and control (*n* = 4) determined by transcriptome sequencing

Gene	GO	Control	Log2 Fold Change	SD	***P***-value
IL6	370.2670351	3.411982453	6.753458123	7.43E-15	1.98E-17
IL18	143.0697496	60.12473955	1.251483826	2.09E-05	6.51E-07
IL1B	72.78808165	8.544040723	3.105534215	7.48E-05	2.87E-06
IL10	26.27799898	2.410378249	3.424663741	0.000228442	1.06E-05
TNF	40.49660233	5.720160668	2.811617316	0.000853265	5.13E-05
IFNγ	6.053708594	0.237173637	4.338547504	0.014835999	0.001836497
IL17B	304.7445786	115.227939	1.402323193	0.021884057	0.003012834

*GO* = Graves' ophthalmopathy; *SD* = standard deviation

## Discussion

GO is a chronic inflammatory disease of the orbital tissues that affects not only orbital adipose tissue, fibroblasts and other connective tissues but also EOMs. EOM enlargement can be seen in patients with GO and is closely related to disease progression. Importantly, autoantibodies targeting EOM antigens have been detected in patients with GO [13, 14], strongly suggesting that EOMs may be a primary target of the autoimmune reaction. Most previous studies focused on the role of orbital adipose tissue and fibroblasts in the formation of GO; only a few studies show the general role of EOMs in the pathogenesis of GO, and these studies do not report the relevance of aberrant epigenetic factors in EOMs to GO. In the current study, we sought to investigate the role of m^6^A methylation in the pathogenesis of GO using surgically excised EOMs.

m^6^A RNA methylation affects a variety of biological processes, especially posttranslational modification, and aberrant RNA methylation has been linked to pathological conditions such as ageing [15], the immune response, the hypoxic stress response [16], tumorigenesis, neurodegeneration and infections. However, the role of m^6^A RNA modification in the pathogenesis of GO has not been shown. In this study, we found that m^6^A RNA methylation levels were significantly increased in EOMs of patients with GO compared with those of control subjects, suggesting a loss of cellular m^6^A homeostasis in EOMs, possibly due to inflammation in the microenvironment. Indeed, we showed that the expression of components involved in m6A RNA modification, such as the m6A RNA methylation writer WTAP and the readers YTHDF2, YTHDC2 and ELF3, was significantly upregulated. Our results suggest that m^6^A RNA methylation might be an important mediator of GO pathogenesis.

Inflammation in the orbit of patients with GO is accepted to be caused by autoimmune responses and inflammatory cytokine release [17, 18]. RNA methylation has been reported to regulate T-cell homeostasis and the inflammatory response by targeting inflammatory pathways [19]. Since we found alterations in global m^6^A RNA methylation and an increase in the activity of the m^6^A methylase complex, demethylases and m^6^A readers in specimens from GO patients, we sought to determine whether there was a potential relationship between abnormal m^6^A RNA methylation and the high expression of inflammation-related genes and activity of their associated pathways. Therefore, RNA-seq analysis was performed, and our results showed that the genes with the most significantly increased expression in GO were those related to the inflammatory response. As shown in Fig. 2a, the data from the transcriptomic landscape of EOMs from patients with GO demonstrated that of the 4393 mRNAs identified, 1047 were upregulated and 1508 were downregulated in GO tissues compared to control tissues. Hierarchical clustering analysis showed that the differences in the gene expression patterns between the GO and control groups were distinguishable. GO enrichment analysis was applied to further validate the data obtained from the transcriptome assay in order to determine whether the specimens were enriched in inflammatory genes. We demonstrated that the top 10 upregulated mRNAs were functionally involved in immune and inflammatory responses, which refers to lymphocyte activation, leukocyte differentiation, cytokine production, cytokine-mediated signaling pathways and the adaptive immune response. In contrast, most of the downregulated genes were related to extracellular matrix (ECM) production and tissue development. The importance of the inflammatory response in GO pathogenesis was further supported by expression analysis of specific inflammatory cytokine genes. As Table 3 shows, the expression of IL-1, IL-6, IL-8, IL-10, IL-17, IFN-γ, and TNF-α was significantly upregulated in EOMs from GO patients. Cytokines such as IL-6, IFN-γ and TNF-α were previously reported to promote fibroblast proliferation and differentiation in GO [17]. More importantly, we found by KEGG analysis of the biological pathways that 12 of 19 pathways were involved in immune and inflammatory responses. Taken together, these results suggest that inflammation is the major pathologic event in the pathogenesis of GO.

Among the proteins regulating m^6^A RNA methylation, WTAP appears to be a regulatory subunit essential for m^6^A methyltransferase activity. In the analysis of the m^6^A regulatory proteins, real-time PCR and RNA-seq analyses showed that the WTAP gene was significantly more highly expressed in the EOMs of patients with GO than in those of control subjects. Increased expression of WTAP has been recognized as a risk factor for a variety of diseases [20]. Functionally, WTAP may be involved in the activation of numerous signaling pathways, such as EGF signaling [21], the mTOR pathway, and Wnt signaling [22], many of these signaling pathways participate in the inflammatory response, implying WTAP might be an important mediator of GO pathogenesis.

In addition to WTAP, other components related to m^6^A RNA methylation identified in our study were YTH domain family members (YTHDFs) and ELF3; the levels of YTHDF1/2 and ELF3 were significantly increased in EOMs from patients with GO. Additionally, the level of another m^6^A binding protein, the hnRNP family protein HNRNPA2B1, was extremely high in EOM specimens from GO patients. The m^6^A binding proteins detected in the EOM specimens might participate in regulating the inflammatory process by targeting the mRNA transcript stability of inflammatory genes [13]. Previous studies revealed that YTHDF2 plays an important role in regulating inflammation through the modulation of NF-κB signaling pathways [23]. Notably, we found that among 19 identified pathways, 12 were related to inflammation. These data suggest a close relationship between YTHDFs and NF-κB signaling pathways in the regulation of the inflammatory response in GO pathogenesis.

Interestingly, we found that the dominated expression of inflammatory genes and its related signaling pathway were associated with downregulation of the gene encoding extracellular matrix in the specimens of EOM. Our result is in tandem with a recent publication, showing that expression of some of the key ECM components such as Collagen 1A1, Collagen 1A2, and Collagen 2A1 were down-regulated in EOM specimens from GO patients although it is a result from protein analysis [24]*.* On the other hand, increased expression of extracellular matrix genes in the specimen of GO patients are seen in earlier reports [25–27]. The difference in terms of ECM gene expression between some of previous studies and ours may be attributed to the following: (1) The methods used for analyzing gene expression are different. In our study, RNA-seq, which is a high-throughput analysis of the whole transcriptome, was utilized to examine gene expression. Its value has been extensively documented in the study of gene expression and function. However, in other reports, PCR or microarray was applied for the evaluation of specific gene expression in specimens of the GO, and thus the focus and baseline are not the same. Therefore, different methods applied in separate studies might result in the variable outcome. (2) Our specimens were collected from the thickest area of the EOM consistently and they accurately represent the pathologic status of the GO. However, in many previous publications, the area of the specimens obtained for the study of the ECM expression have not been defined [17, 24, 28]. Since gene expression may be variable in the tendon and the muscle component of GO specimens [29], this factor may influence the expression of the ECM gene. (3) The different result may be due to the specimens obtained from the different statuses of GO: an active inflammatory status (inflammatory cytokines production) or a static status (connective tissue growth and ECM deposition) [30]. Inflammation dominates the pathologic process of active GO [31]. Pro-inflammatory factors such TNF, IL-1, IL-6, IL-8 are much higher in the specimens of active status GO than the non-active status. Hiromatsu indicate that increased expression of TNF in EOM was proportional to the enlargement of EOM [28]. Also, variable accumulation of extracellular matrix is dependent on the enzyme of MMPs which can degrade ECM; the increased levels of MMPs were seen in the inactive phase of GO patients [32]. Importantly, inflammatory cytokines can upregulate the MMPs, including the cell of orbital fibroblasts [33–35]. In our study, the expression of the inflammatory genes especially IL-6, IL-18, and TNF (see Table 3) and their signaling pathways were dominating events, implying an active status of the GO. Therefore, this might be one of the reasons that fewer ECM-related genes were seen in our study. (4) The deregulation of m^6^A methylation may be related to the downregulation of ECM in GO specimens. In this study, the increased m^6^A methylation was associated with the downregulation of ECM in the GO specimen, which was consistent with Yu et al.’s study that showed that increased m^6^A methylation may result in the decreases of the expression of collagen which is a major component of ECM [36]. We found that the significantly enhanced transcript of WTAP (as components of the mammalian m6A methyltransferase complex) was associated with downregulation of ECM genes in GO specimens. Taken together, it seems plausible that the expression of ECM genes in GO specimens may be regulated by RNA methylation [36–38]. In fact, WTAP might especially contribute to the decreased expression of ECM genes [37, 39]. Certainly, additional research will be required to confirm this finding in the future.

## Conclusion

In summary, our study showed that the total m^6^A content was significantly increased in GO patients and identified aberrant expression of m6A methylation regulators. Importantly, we demonstrated that most of the upregulated genes and biological pathways in specimens from GO patients were related to the immune response and inflammatory processes. Our study provides new insights into the pathogenesis of GO and demonstrates that targeting m^6^A methylation may be a potential therapeutic approach for GO.

## Data Availability

The data that support the findings of this study are available upon reasonable request from the corresponding author.
